# How Validation Methodology Influences Human Activity Recognition Mobile Systems

**DOI:** 10.3390/s22062360

**Published:** 2022-03-18

**Authors:** Hendrio Bragança, Juan G. Colonna, Horácio A. B. F. Oliveira, Eduardo Souto

**Affiliations:** Institute of Computing, Federal University of Amazonas, Manaus 69067-005, Brazil; hendrio.luis@icomp.ufam.edu.br (H.B.); juancolonna@icomp.ufam.edu.br (J.G.C.); horacio@icomp.ufam.edu.br (H.A.B.F.O.)

**Keywords:** human activity recognition, validation methodology, leave-one-subject-out cross-validation, explainable methods, Shapley additive explanations, machine learning

## Abstract

In this article, we introduce explainable methods to understand how Human Activity Recognition (HAR) mobile systems perform based on the chosen validation strategies. Our results introduce a new way to discover potential bias problems that overestimate the prediction accuracy of an algorithm because of the inappropriate choice of validation methodology. We show how the SHAP (Shapley additive explanations) framework, used in literature to explain the predictions of any machine learning model, presents itself as a tool that can provide graphical insights into how human activity recognition models achieve their results. Now it is possible to analyze which features are important to a HAR system in each validation methodology in a simplified way. We not only demonstrate that the validation procedure *k*-folds cross-validation (*k*-CV), used in most works to evaluate the expected error in a HAR system, can overestimate by about 13% the prediction accuracy in three public datasets but also choose a different feature set when compared with the universal model. Combining explainable methods with machine learning algorithms has the potential to help new researchers look inside the decisions of the machine learning algorithms, avoiding most times the overestimation of prediction accuracy, understanding relations between features, and finding bias before deploying the system in real-world scenarios.

## 1. Introduction

Human Activity Recognition (HAR) is an emergent research area for autonomous and real-time monitoring of human activities and has been widely explored because of its good practical applications, such as behavior detection, ambient assisted living, elderly care, and rehabilitation [1,2,3,4,5,6,7,8]. Individuals express their routines through activities that are performed in particular situations and understanding these situations enables people to improve their daily lives. Physical activities performed by an individual (e.g., walking and running) can create recommendations and avoid the negative impacts of illness. For instance, the time spent sitting is associated with an increased risk of becoming obese and developing diabetes and cardiovascular diseases [3]. A HAR system can observe elderly people by analyzing data from a smart wearable and improve their lifestyle by warning them about forthcoming unprecedented events such as falls or other health risks [9].

Smartphones have been used to monitor everyday activities automatically through a variety of embedded sensors such as accelerometers, gyroscopes, microphones, cameras and GPS units [1,10]. Understanding how individuals behave by analyzing smartphone data through machine learning is the fundamental challenge in the human activity recognition research area [11].

To recognize physical activity from reading data from sensors, most proposed solutions rely on the Activity Recognition Process (ARP) protocol: Data acquisition, pre-processing/segmentation, feature extraction, classification and evaluation [10,12,13,14]. Several parameters in each one of these stages (sample size, experimental methodology, cross-validation settings and type of application) can affect the overall recognition [15]. Even when these parameters are well adjusted, the final evaluation of the system may not reflect the true accuracy when recognizing data from new individuals. The main reason for this is that, in most cases, the methodology used to validate the results does not consider the label that identifies the individuals.

The most commonly adopted validation strategy in Machine Learning (ML) literature is the *k*-fold cross-validation (*k*-CV) [16]. The *k*-CV splits a dataset into two subsets: One for training the ML algorithm and one for testing the performance, repeating this process *k* times. The *k*-CV procedure does not consider whether all samples belong to the same subject (i.e., individual). This is usually because of the windowing step used to segment the time series during the pre-processing stage. Therefore, in a HAR application that aims for generalization, randomly partitioning the dataset becomes a problem when samples of one subject are in both training and test sets at the same time. As a result, an information leak appears, artificially increasing the classifier’s accuracy. We can confirm this observation in several studies in the literature [7,17,18,19,20,21,22].

In practice, the introduction of illegitimate information in the evaluation stage is unintentional and facilitated by most data partitioning processes, making it hard to detect and eliminate. Even then, identifying this situation as the reason for the overestimated results might be non-trivial [18,23].

In this article, we use the explainable artificial intelligence (XAI) tools’ capacity to detect and address bias and fairness issues when choosing different validation methodologies. This is a critical topic that has grown rapidly in the community because the decisions of machine learning models can reproduce biases in historical data used to train them [24]. A variety of reasons, like lack of data, imbalanced datasets and biased datasets, can affect the decision rendered by the learning models. We found it is possible to explain model behavior and its capability in a simple way. Machine learning engineers can use this information to suggest modifications needed in the system to reduce critical issues linked to bias or fairness.

Our work aims to discover bias problems that overestimate the predictive accuracy of a machine learning algorithm because of an inappropriate choice of validation methodology. We examine how different HAR system approaches make generalizations based on a new subject(s) by using *k*-fold cross-validation, holdout and leave-one-subject-out cross validation. In particular, we show how the SHAP (Shapley additive explanations) framework presents itself as a tool that provides graphical insights into how human activity recognition models achieve their results. This is important because it allows us to see which features are relevant to a HAR system in each validation method.

We can summarize the main contributions of this work as follows:

(1) We evaluate three different approaches for building a HAR system: Personalized, universal and hybrid. Our experiments reveal pitfalls caused by incorrectly dividing the dataset, which can lead to unnoticed over-fitting. We show that *k*-CV achieves an average accuracy of 98% on six human activities, whereas with leave-one-subject-out cross-validation the accuracy drops to 85.37%. We achieved the results by merging three widely used datasets, SHOAIB [2], WISDM [25] and UCI-HAR [26], which have human activities performed by 59 different subjects.

(2) We propose a new approach by using XAI methods to show how machine learning models choose different features to make its prediction based on the selected validation strategy. We performed several experiments that allowed us to measure the impacts of each of these methodologies on the final results. With this, we could quantify the importance of choosing the correct evaluation methodology of a HAR system.

The remainder of this paper is organized as follows. Section 4 presents the most common procedures for building a HAR system. Section 5 presents a discussion of a fair evaluation for HAR systems. Section 6 introduces explainable algorithms used to interpret the predictions of machine learning models. Section 7 presents the experimental protocol and Section 8 the results of our evaluation scenarios. Section 3 presents the work related to this research. Finally, Section 9 presents the conclusions of this work.

## 2. Human Activity Recognition

Smartphones are devices capable of monitoring everyday activities automatically through a variety of built-in sensors such as accelerometers, gyroscopes, microphones, cameras and GPS units [10]. Human activity recognition involves complicated tasks which often require dedicated hardware, sophisticated engineering and computational and statistical techniques for data pre-processing and analysis [7].

**Figure 1:**
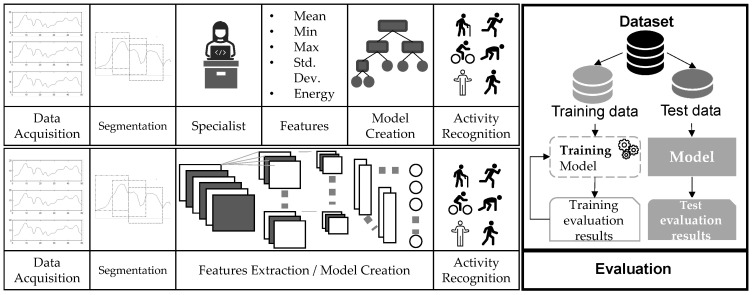
The common methodology used in HAR: Data acquisition, segmentation, feature extraction, classification and evaluation.

To find patterns in sensors data and associate them to human activities, the standard pipeline used in most works follows the Activity Recognition Process (ARP) protocols [7,11,13,27,28,29,30]. As depicted in Figure 1, ARP consists of five steps, acquisition, preprocessing, segmentation, feature extraction and classification [7]. Our work also includes the evaluation phase in the ARP pipeline to present in detail how validation methodology impacts the general performance of a HAR system. We can find in literature extensions of the standard pipeline with specific stages such as annotation and application stage [31], or even privacy and interpretability [32,33].

### 2.1. Data Acquisition

In the data acquisition phase, motion sensors are used to gather data, such as angle, vibration, rotation and oscillation from the smartphone. The individual actions are reflected in the sensor data or linked to the physical environment in which the device is located. For this reason, choosing suitable sensors is crucial. Currently, the accelerometer sensor is mostly used in HAR systems because it is built-in in most smartphones and wearable devices and also has shown superior results concerning representing activities if compared with other inertial sensors. The combination of accelerometer and gyroscope allows HAR systems to find patterns in the sensor signals and associate them with the activities performed, such as activity of daily living (ADL) and sports. However, finding such patterns is not trivial, since smartphones are often held near different parts of the user’s body and each subject may have a personal signature of activity [11,13,28].

### 2.2. Pre-processing and Segmentation

After data acquisition, the raw data collected by motion sensors may contain noises and must be processed, adapted into a readable format and segmented to be used by future stages of the HAR applications. The segmentation phase comprises dividing the signals that are recorded continuously into smaller segments. Choosing smaller segments allows the detection of activities faster, since the wait to mount the segment is smaller and the resource requirements in the process are also reduced. Using larger segments allows more complex activities to be recognized, but an additional amount of time will be required to assemble and process the segment. The HAR community have used different sizes of segments in the literature with the use of segment sizes ranging from 1 s to 10 s, with a recognition rate above 90% [7,25,34,35].

### 2.3. Feature Extraction Process

The feature extraction phase aims to find a high-level representation from each activity segment. For sensor-based activity recognition, feature extraction is more difficult because there is inter-activity similarity. Different activities may have similar characteristics (e.g., walking and running). Therefore, it is difficult to produce distinguishable features to represent activities uniquely [32].

Many HAR systems are based on shallow approaches. Features are handcrafted by a domain specialist that transform data gathered from sensors into a high-level representation. The handcraft features can be divided into three domains: Time, frequency and symbolic [11,13,14,26,27,35].

The time-domain features are obtained by simple statistical calculations, such as average, median and standard deviation. These features are simple to calculate and understand and have low computational complexity when compared to other feature extraction processes, such as those based on deep neural networks [12,36]. The frequency-domain features are used to capture natural repetitions by decomposing the time series into a set of real and imaginary values representing wave components, through the use of Fourier or Wavelet transforms, for example. The symbolic domain features represent the sensor signals in a sequence of symbols obtained through a discretization process, allowing data to be compressed into a smaller space than the original data [11,27].

The low computational complexity and its simple calculation process make hand-crafted features still practicable. The major disadvantage is that resources created or selected manually are time consuming, domain specific and require specialized knowledge.

### 2.4. Human Activity Classification

A machine learning algorithm can automatically detect patterns in a dataset and can be used to make decisions in situations of uncertainty. There are several supervised learning algorithms such as decision tree, naive Bayes, support vector machine (SVM), artificial neural networks (ANN), logistic regression and KNN (K-Nearest Neighbors) [37]. For these methods, it is essential that the sensors data be converted into a high-level representation, since machine learning models do not work very well if they are applied directly to the raw data [25].

More recently, deep learning models reach human-level performance in various domains, including HAR. This approach can automatically learn abstract features from sensors’ data and thus eliminating the need for a dedicated feature extraction phase because the entire process is performed within the network hidden layers. Moreover, it outperforms in performance when applied to large masses of data if compared with traditional ML algorithms.

For HAR, the most common solutions found in the literature are based on Convolutional Neural Networks (CNN) and Long-Short-Term Memory Recurrent (LSTMs) [28,36,38,39]. Unfortunately, one of the main drawbacks of deep learning algorithms is related to their high computational cost which could make its implementation unsuitable for creating real-time HAR applications implemented on devices with low computational power [36,39].

A crucial stage of the classification process is to assess the performance of the trained machine learning algorithm. The next section presents the most common evaluation metrics used to estimate the future performance of a classifier.

### 2.5. Evaluation Metrics for HAR Systems

The performance of the classification model is evaluated by a set of metrics that shows how reliable is the model under evaluation, in mathematical terms [16,40]. The evaluation metrics commonly used in the smartphone-based HAR literature are accuracy, recall (sensitivity), specificity, precision and *F*-measure [15,41]. In HAR, accuracy is the most popular and can be calculated by dividing the number of correctly classified activities and the total number of activities. Accuracy gives a general idea of classification models’ performance. However, this metric treats all classes as equally important in a dataset. This leads to an unreliable metric in unbalanced databases, strongly biased by dominant classes, usually the less relevant background class [15,42]. To avoid unreliable results in unbalanced datasets, there are other metrics that evaluate classes separately, such as precision and recall, as shown in Table 1.

The precision metric is the ratio of true positives and the total positives. If the precision value is equal to "1", it means that the classifier correctly predicts all true positives and is able to correctly classify between correct and incorrect labeling classes. The recall metric analyzes the true positive (*TP*) rate to all the positives. A low recall value means that the classifier has a high number of false negatives. Finally, *F*-measure deals with a score resulting from the combination of precision and recall values to provide a generic value that represents these two metrics. High *F*-measure values imply both high precision and high recall. It gives a balance between precision and recall, which is suitable to imbalanced classification problems, including HAR.

Different from most works that comprise all ARP stages, our focus in this article relies on the evaluation process and validation methodologies. By looking deep into the evaluation stage, we aim to understand how human activity recognition models achieve their results according to validation methodology.

## 3. Related Works

Many works in the literature alert researchers to the correct assessment of activity recognition models and, although this problem is widely known, it is often overlooked. Hammerla and Plötz [43] found inappropriate use of *k*-CV by almost half of the retrieved studies in a systematic literature review that used accelerometers, wearable sensors or smartphones to predict clinical outcomes, showing that record-wise (segmentation over the same user data) cross-validation often overestimates the prediction accuracy. Nevertheless, HAR system designers often either ignore these factors or even neglect their importance. Widhalm et al. [22] also has pointed unnoticed over-fitting because of autocorrelation (i.e., dependencies between temporally close samples). Hammerla and Plötz [43] showed that the adjacent overlapping frames probably record the same activity in the same context and, therefore, they share the same information. These adjacent segments are not statistically independent.

Dehghani et al. [29] extend the work of Banos et al. [44] by investigating the impact of Subject Cross-Validation (Subject CV) on HAR, both with overlapping and with non-overlapping sliding windows. The results show that *k*-CV increases the classifier performance by about 10%, and even by 16% when overlapping windows are used. Bulling et al. [15] provide an educational example, demonstrating how different design decisions in the HAR applications impact the overall recognition performance.

Gholamiangonabadi et al. [45] examine how well different machine learning architectures make generalizations based on a new subject(s) by using Leave-One-Subject-Out (LOSO) in six deep neural networks architectures. Results show that accuracy improves from 85.1% when evaluated with LOSO to 99.85% when evaluated with the traditional 10-fold cross-validation.

In contrast to the reviewed works related that deal with validation methodologies, our study examines bias problems that overestimate the predictive accuracy of a machine learning algorithm using graphical insights obtained from SHAP framework to understand how human activity recognition models achieve their results according to validation methodology. While there are works that make use of explainable methods in HAR context [9,33,46,47], most of the methods for explainability focus on interpreting and making the entire process of building an AI system transparent. The finds focused on validation methodology are important because it allows us to see which features are relevant to a HAR system in each validation method. We examine how different HAR systems approaches make generalizations based on a new subject(s) by using *k*-fold cross-validation, holdout and leave-one-subject-out cross-validation.

## 4. Evaluation Procedures

A common practice for computing a performance metric (e.g., accuracy), when performing a supervised machine learning experiment, is to hold aside part of the data to be used as a test set [16]. Splitting data into training and test sets can be done using various methods, such as hold-out, *k*-fold cross-validation (*k*-CV), leave-one-out cross-validation (LOOCV) and leave-one-subject-out (LOSO). Then, the classifier is trained on the training set, while its accuracy is measured on the test set. Thus, the test set is seen as new data never seen by the model before [16]. We briefly explained these methods in the following sections.

### 4.1. Hold-Out

The hold-out is the simplest form of splitting data and relies on a single split of the dataset into two mutually exclusive subsets called training set and a test set [16,42]. A common dataset split uses 70% or 80% for training and 30% or 20% for testing. The advantage of this method is the lower computational load. The hold-out is a pessimistic estimator because the classifier is trained only with part of the samples. If more data is left for the test, the bias of the classifier will be higher, but if only a few samples are used for the test, then the confidence interval for accuracy will be wider [42]. It has lower computational costs because it needs to run once but if the data are split again, the results of the model probably will change. This means that the accuracy depends on the subject(s) selected for the evaluation [45].

### 4.2. K-fold Cross-Validation (k-CV)

The *k*-CV consists of averaging several hold-out estimators corresponding to different data splits [16,37,40]. This procedure randomly divides the dataset (from one subject or all subjects) into *k* disjoint folds with approximately equal size, and each fold is in turn used to test the classification model induced from the remaining k−1 folds. Then, the overall performance is computed as the average of the *k* accuracies resulting from *k*-CV [40,42]. The disadvantage of using this strategy is its computational cost when the values of *k* are relatively high for large samples. In addition, no single cross validation procedure is universally better but it should focus on the particular settings [16].

### 4.3. Leave-One-Subject-Out Cross-Validation (LOSO)

The LOSO (Leave-One-Subject-Out Cross-Validation) strategy aims at finding out whether a model trained on a group of subjects generalizes well to new, unseen subjects. It is a variant of the *k*-fold cross-validation approach but with folds consisting of a subject [45]. To measure this, we have to ensure that all of the samples in the test fold come from subjects that are not represented at all in the paired training fold.

The LOSO strategy uses a subset of size *p* for testing and n−p for training, where *p* keeps all of the samples from a single subject together. This procedure ensures that the same subject is not represented in both testing and training sets at the same time. This configuration allows evaluating the generalization of the model based on data from new subjects; otherwise, if the model learns person-specific features, it may fail to generalize to new subjects.

When the number of subjects in a dataset is small, it is common to adopt LOSO to evaluate the performance of a classification algorithm. The LOSO is an extreme case of *k*-CV, where the number of folds is equal to the number of subjects on the dataset. It has a high variability as only one subject is used as the validation set to test the model prediction. This exhaustive procedure should be used when the random partition in *k*-CV has a large impact on performance evaluation [40,42].

### 4.4. Types for HAR Systems

The main goal of machine learning algorithms is to develop models that work not only on the specific dataset for which they were trained but also on new and unseen data. However, what does new data mean in human activity recognition problems? To answer this question, we need to know the purpose for which the algorithm is being developed. If we developed specifically for one subject, new data means new samples, or records, from the same subject. This falls into the category of personal systems. However, if the goal is to develop universal systems that can classify activities from a new subject, new data means new subjects.

**Figure 2:**
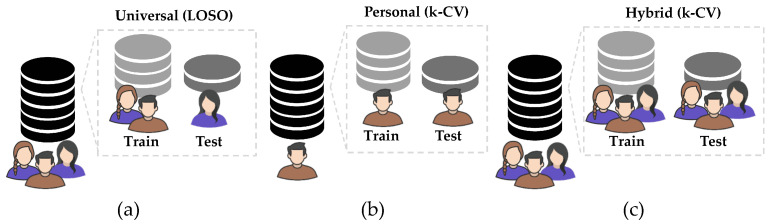
Visualization of each procedure used to build the model types for HAR systems. To build universal models, the LOSO procedure (**a**) separates train and test by subjects. For personalized models (**b**), the *k*-CV is used with samples of only one subject. Finally, for hybrid models (**c**), the *k*-CV is also used, but in this case, for a group of subjects, data are split by samples into the training and test set.

Each type of HAR system addresses a slightly different learning problem and makes different assumptions about how the learning algorithm is applied [45,48]. There are three types of HAR systems [45,49,50], as shown in Figure 2: Universal or generalized, personal or personalized and hybrid.

Universal systems must be capable of generalizing patterns of any subject. The most common validation procedure used in this context is the leave-one-subject-out cross-validation (LOSO) [45,51]. The LOSO considers the subject information when splitting the training and test set. This information is useful for preventing data from the same subject being present in both sets, as shown in Figure 2a.

Personalized systems aim at creating models that are experts in recognizing patterns from the same subject. This is called personalized validation [8,34,49,50,52]. In personalized systems, the selected machine learning algorithm is trained and tested with data from only one subject. Thus, the samples from this subject are divided between training and testing, as shown in Figure 2b.

Most studies in HAR use hybrid systems to validate the model performance with *k*-CV as validation methodology. It is hybrid because all sample data from over one subject are mixed and data from the same subject can be in the training and test sets, as shown in Figure 2c [25].

Each system has a specialization and this determines how training and test data are partitioned for evaluation. The next section presents a discussion about the correct evaluation that systems designers should consider in each model type.

## 5. A Fair Evaluation for Human Activity Recognition Systems

Most machine learning algorithms need Independent and Identically Distributed (i.i.d.) data [40]. If the recruitment process used is i.i.d., the subjects will be a representative sample of the overall population. Otherwise, if the i.i.d. is not assured, such as recording activities in which several samples are collected from each subject, the sampling process might generate groups of dependent samples. Therefore, *k*-CV is not a good procedure for validating universal models because of the temporal dependency among samples from the same subject. This means that the model trained using *k*-CV procedure knows the activity patterns of a specific subject, shared in both training and test sets. This situation may lead a model to learn a biased solution, where the machine learning algorithm can find a strong association between unique features of a subject (e.g., walking speed), artificially increasing its accuracy on the test set [20,45]. It explains why some systems report high accuracies.

To minimize the problem of weak generalization, the data should be adequate for a fair validation procedure according to the application purpose. This means that the application of the *k*-CV to new samples or new subjects does not measure the same thing and it should be determined by the application scenario, not by the statistics of the data. For instance, if the generative process has some kind of group structure, such as samples collected from different subjects, experiments or measurement devices, it is more appropriate to use cross-validation by subject or by a group. In this procedure, the preservation of independence means that full subjects’ information must be left out for CV. For applications that aim at a personalized classification model, the traditional *k*-CV is an acceptable procedure.

## 6. Explainable Algorithms for Creating Fairer Systems

Explaining the decisions made by a machine learning model is extremely important in many applications. Explainable models can provide valuable information on how to improve them and also help to better understand the problem and the information provided by the input variables [53].

Identifying issues like biased data could allow systems design to select sensitive attributes that they want to focus their evaluations on. This is a key feature for explainability that has a clear purpose for evaluating fairness, as well as in non-fairness-related explanations where certain features should be weighed more or less heavily in class selection than others. Mitigating the bias and unfairness within the training data is a necessity, both out of ethical duty and because of the impact that perceived inaccuracies have on user trust [54,55,56].

More recently, XAI methods have been proposed to help interpret the predictions of machine learning models, as example, LIME [57], Deep Lift [58] and SHAP [59]. XAI methods have been used in HAR context to understand the rationale behind the predictions of the classifier [9,33,46,47]. In this work, we choose a unified framework for interpreting model predictions, called SHAP (Shapley additive explanations), to explain graphically and intuitively the results of different validation methodologies used in HAR systems.

The SHAP (Shapley additive explanations) [59] is based on a game-theoretic approach extensively used in literature to explain the predictions of any machine learning model. The Shapley values acted as a unified measure of feature importance. It aims to explain the prediction of an instance *x* by computing the contribution of each feature to the prediction. In summary, the Shapley values give each feature a score that is distributed across the features of that instance.

The Algorithm 1 shows the pseudo-code to approximate Shapley estimation for single feature value [60]. First, select an instance of interest *x*, a feature *j* and the number of iterations *M*. For each iteration, a random instance *z* is selected from the data and a random order of the features is generated. Two new instances are created by combining values from the instance of interest *x* and the sample *z*. The instance $x_{+j}$ is the instance of interest, but all values in the order after feature *j* are replaced by feature values from the sample *z*. The instance $x_{-j}$ is the same as $x_{+j}$, but in addition has feature *j* replaced by the value for feature *j* from the sample *z*. The difference in the prediction from the black box is computed by $\phi_{j}^{m}=\hat{f}\left(x_{+j}^{m}\right)-\hat{f}\left(x_{-j}^{m}\right)$ and all differences are averaged, resulting in $\phi_{j}\left(x\right)=\frac{1}{M}\sum_{m=1}^{M}\phi_{j}^{m}$. Averaging implicitly weighs samples by the probability distribution of *X*. The procedure has to be repeated for each of the features to get all Shapley values.
**Algorithm 1** SHAP basic algorithm1:**Required**: **M**: Number of interactions; **x**: Instance of interest; **j**: Features index; **X**: Data matrix; **f**: Machine learning model.2:**procedure**SHAP(M,x,j,X,f)3:    **for** m=1 in M **do**4:        Draw random instance z from the data matrix X5:        Choose a random permutation o of the feature values6:        Order instance x: $x_{0}=\left(x_{\left(1\right)},...,x_{\left(j\right)},...,x_{\left(p\right)}\right)$7:        Order instance z: $z_{0}=\left(z_{\left(1\right)},...,z_{\left(j\right)},...,z_{\left(p\right)}\right)$8:        Construct two new instances9:        Compute marginal contribution $\phi_{j}^{m}=\hat{f}\left(x_{+j}\right)-\hat{f}\left(x_{-j}\right)$10:    **end for**11:    Compute Shapley value as the average $\phi_{j}\left(x\right)=\frac{1}{M}\sum_{m=1}^{M}\phi_{j}^{m}$12:**end procedure**

The Shapley values can be combined into global explanations [59,60] by running SHAP algorithm for every instance to obtain a matrix of Shapley values, one row per data instance and one column per feature. We can interpret the entire model by analyzing the Shapley values in this matrix. The idea behind SHAP feature importance is simple: Features with large absolute Shapley values are important. Since we want the global importance, we average the absolute Shapley values per feature across the data.

In this sense, SHAP framework can understand the decision-making of a classification model globally by summarizing how a model generates its outputs. Global explanations are beneficial as they might reveal biases and help diagnose model problems [61]. They can also explain model predictions at the instance level once each observation gets its own set of SHAP values. This greatly increases its transparency.

We have used a specific method for local explanations of tree-based models, called TreeExplainer [59,62], which provides fast and accurate results by calculating the SHAP values for each leaf of a tree.

## 7. Experimental Protocol

This section describes the experimental protocol, considering four evaluation scenarios. We detail the datasets used in this study, the baselines that are built with time and frequency domain features and the performance metrics.

### 7.1. Datasets

The physical activity data used in this work were obtained from three publicly available datasets: SHOAIB (SH) [2], WISDM [25] and UCI [26]. Table 2 presets a summarization of the datasets used in our study [11].

In our experiments, we use only accelerometer data. For the WISDM dataset, we chose users who performed all activities, totaling 19 individuals. For the SHOAIB dataset, we selected the six most similar activities with WISDM and UCI datasets, so that all datasets had the same number of classes to compare results. We removed the Jogging and biking activities from our experiments because of this. The SHOAIB dataset contains data collected from five different body positions merged to run our experiments. Moreover, SHOAIB is balanced and should represent a fairer evaluation. This means a reduction in bias caused both by individuals with more activity or unbalanced class labels.

### 7.2. Baselines

The baselines are shallow approaches based on traditional machine learning algorithms such as Random Forest (RF), Naive Bayes (NB), K-Neighbors (KNN) with *k* = 1 and Simple Logistic (SL). We trained each algorithm with a set of handcraft features extracted from the time and frequency domain. Table 3 presents a list of mathematical functions used for creating the features used by the baselines [7,11,27]. The experiments were executed in the WEKA library (Waikato Environment for Knowledge Analysis) [63].

While we know the benefits of using complex models, especially in dealing with large masses of data, in our HAR context, we are adopting simple models, such as random forest, mainly because of speed, good performance and easy interpretation. Our focus is not on evaluating the best model for recognizing human activities but discovering bias problems that overestimate the predictive accuracy because of an inappropriate choice of validation methodology.

### 7.3. Evaluation Scenarios

The experiments are based on the three model types commonly found in literature: Personalized, universal and hybrid.

1. Universal model: This scenario evaluates the model’s generalization capacity using LOSO. We separated the data set in training and testing and there is a guarantee that these two sets are not mixed.
2. Personalized model: This scenario evaluates the model personalization capacity using *k*-CV. We partition the data from a single subject into two sets of samples. The validation process is based on 10-CV.
3. Hybrid model: This scenario evaluates a hybrid model that combines all subject data from the universal model using the validation process of the personal model.

In addition, we use SHAP explanation method to understand how machine learning models tend to select different features based on the validation methodology. For this experiment, we have used UCI dataset with a 561-feature vector with time and frequency domain variables [26] using holdout and cross-validation to analyze how model select different features to make its predictions. We select the Random forest algorithm to conduct this experiment. In Section 8.1 we also provide explanations of individual predictions using shap values based on subject number 9 of UCI dataset.

### 7.4. Performance Metrics

To measure the performance metrics of universal, hybrid and personal models, we use standard metrics such as accuracy, precision, recall and F-measure (or F-Score) [1,45] obtained from confusion matrix analysis. We used other metrics, besides accuracy, since it alone may not be the most reliable way to measure the actual performance of a classification algorithm, mainly because class imbalance can influence the results. Our research employs the metrics summarized in Table 1 (Section 2).

## 8. Results

**Figure 3:**
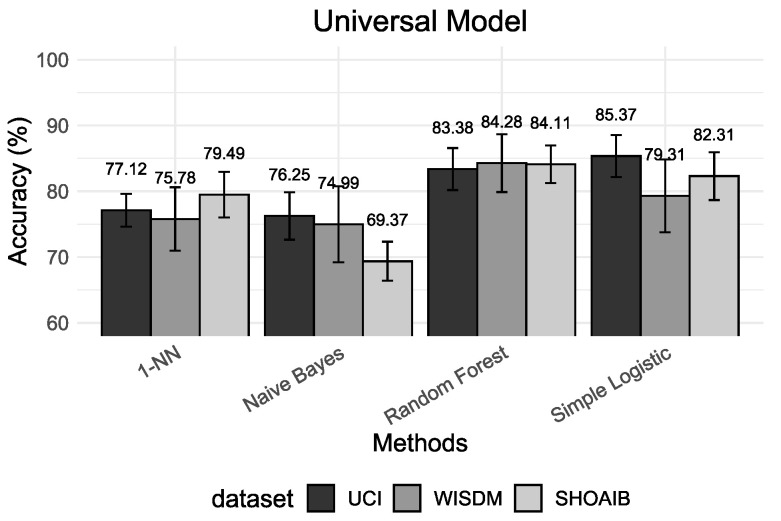
Accuracy results based on universal model for the classifiers 1-NN, Naive Bayes, Random Forest and Simple Logistic.

**Figure 4:**
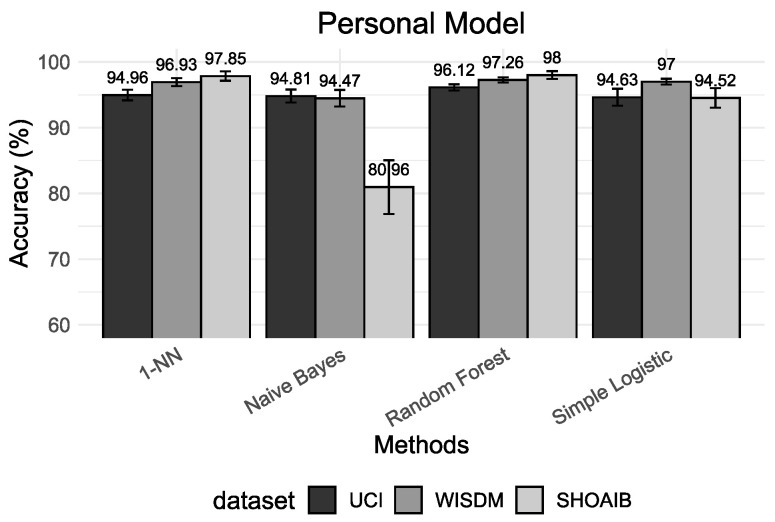
Accuracy results based on personalized model for the classifiers 1-NN, Naive Bayes, Random Forest and Simple Logistic.

**Figure 5:**
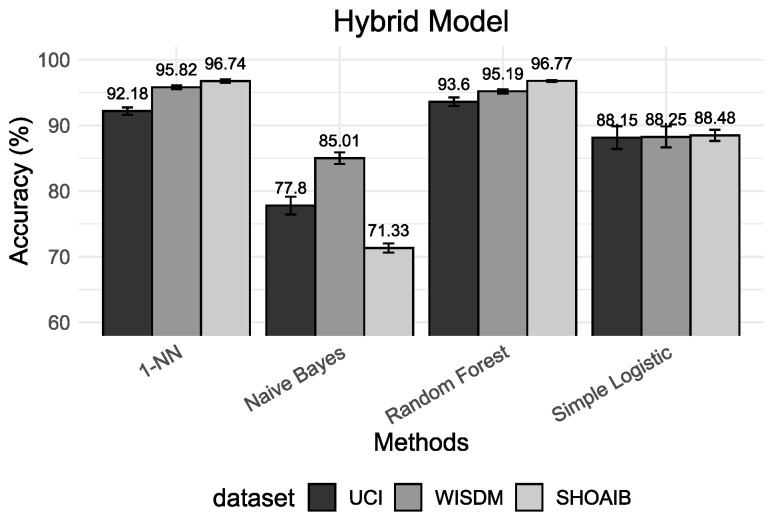
Accuracy results based on hybrid model for the classifiers 1-NN, Naive Bayes, Random Forest and Simple Logistic.

This section presents a comparative analysis of different validation procedures based on the machine learning results and the interpretable methods. We divide results into four different setups. First, we deal with the validation of personalized models. Second, we deal with the valuation of universal models. The third scenario deals with the validation of a hybrid model. The performance results of five classifiers are presented using accuracy as the main metric for universal models (Figure 3), personalized models (Figure 4) and hybrid models (Figure 5). Finally, we present the insights based on Shapley values that give us an alternative manner to analyze and understand results.

The results presented in Figure 3, Figure 4 and Figure 5 show that, for all classification algorithms, the personal models perform very well, the hybrid models perform similarly and the universal models have the worst performance. The main reason for this result is that different people may move differently and universal models cannot effectively distinguish between some activities which are highly related to the respective user and, consequently, it will have low performance on classification and a high confidence interval because of the variance in the population.

The hybrid models have performed much closer to personal models. Most HAR studies use cross-validation (*k*-CV) to evaluate and compare algorithms’ performance. The mixture between the train and test sets results in a classification model that already knows part of its test set. In other words, the model trained using the *k*-CV can associate activity patterns of a specific subject on both train and test sets. The result of this process is the creation of models with higher classification accuracy. However, they do not reflect reality. If we insert new subjects into the domain, the model will have difficulties recognizing them.

Table 4 and Table 5 analyze the performance of the best model (random forest) on individual subjects for universal and personalized scenarios using SHOAIB dataset. In Table 4 the rows for subjects 1 to 10 represent the folds of the subject cross-validation. For subject 1 row, the model is trained using subjects 2–10 and evaluated on subject 1 and so on. In Table 5 the rows represent subjects 1 to 10. For subject 1 row, the model is trained using only data of subject 1, subject 2 using data of subject 2 and so on.

As can be observed in Table 4 the accuracy of the same model varies greatly among subjects as opposed to *k*-CV used in the personalized model (Table 5) in order to capture variability among subjects. Moreover, the standard deviation is higher for the universal model than for the personalized model. This shows that if a single generic model will be used for all users, the standard deviation should be considered when selecting the model.

**Figure 6:**
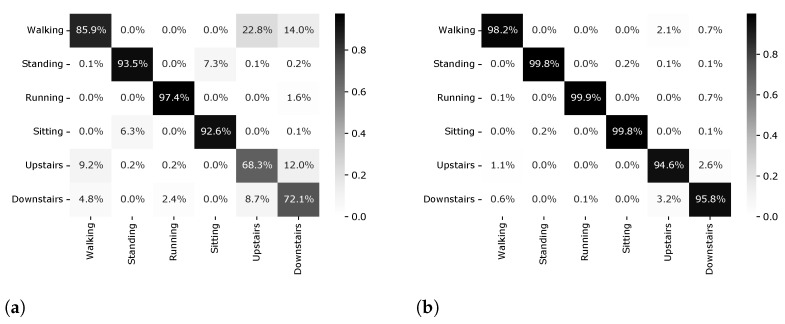
Confusion matrix of Random Forest algorithm results for universal model and personal model using the SHOAIB dataset. (**a**) Universal model. (**b**) Personalized model.

The Figure 6 allows us to compare the universal model (LOSO) and personal model (*k*-CV) using the Random Forest algorithm. As it can be noticed from the confusion matrix, most of classes are correctly classified with very high accuracy. However, the universal model has difficulty in differentiating the walking class from upstairs and downstairs classes. This is expected as these three are very similar activities so the underlying data may not be sufficient to accurately different them. For stationary classes, such as standing and sitting, the misclassification is very low, demonstrating that distinction between those classes generalizes well for new subjects.

For methods whose goal is to generate a custom classification model, such as a personalized or hybrid model, *k*-CV will work very well. However, it may not be a good validation procedure for universal models. These results have shown the importance of a careful analysis of these different scenarios.

### 8.1. Global Explanations Using Shap Values

In this section, we showed through a summary plot [59,60] how validation methodologies affect feature importance and also discuss strategies to avoid potential issues in pre-processing data.

The summary plot combines feature importance with feature effects, considering the absolute average Shapley values along with all the classes. With a multi-classification problem, it shows the impact of each feature considering the different classes. The position on the y-axis is determined by the feature and on the x-axis by the Shapley value.

**Figure 7:**
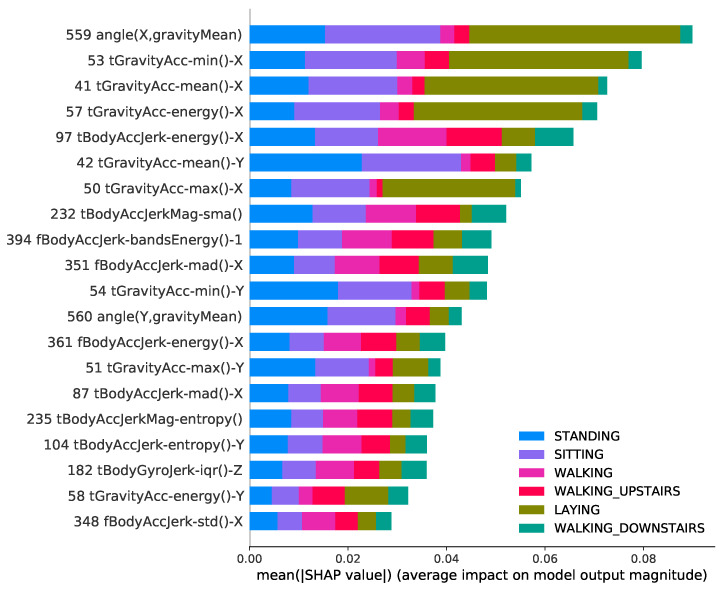
Summary plot for SHAP analysis using holdout methodology on UCI dataset. It shows the mean absolute SHAP value of 20 most important features for six activities.

**Figure 8:**
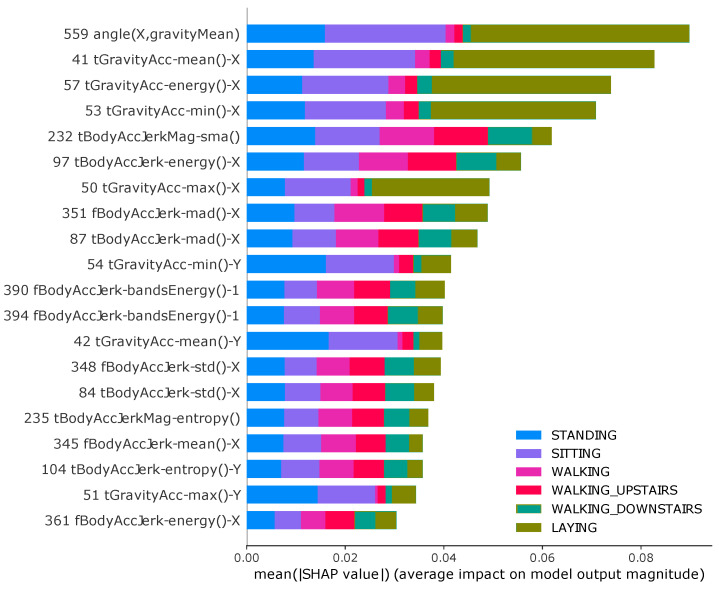
Summary plot for SHAP analysis using cross-validation on UCI dataset. It shows the mean absolute SHAP value of 20 most important features for six activities.

Figure 7 and Figure 8 show that there are changes in the features that each model chose based on the validation methodology. This slightly different importance that the classification model gives for features when using different validations methodology causes a great boost in performance for cross-validation. Moreover, the CV has 17 features in common with holdout, a difference of 15%.

Given a feature, we also extract the importance proportion for each class. The results also show that the importance that each feature assigns to the classes is different according to the adopted methodology. By analyzing the contribution of each class for each feature, for example, Feature 53 (related with accelerometer gravity in x-axis) has a greater contribution to the class "walking upstairs" in the holdout methodology while it contributes more to the class “laying” when using the CV. Similar results can be observed in features 97, 57, 41 and many others.

When using cross-validation, the classifier already knows the individual attributes because its data can be shared in training and testing. Knowing the individual’s pattern, the classifier can choose features that best suit the individual’s behavior. Models trained using different training and test sets are more realistic because they reflect the approximate performance of what would happen in the real world, and thus it is possible to choose classifiers that select more generic features that better represent the population or group of individuals.

While the results presented are promising, in many situations, it is not trivial to find meanings in statistical features extracted from inertial sensors in HAR. However, our results show that the adopted methodology can significantly influence the selection of features to overestimate the results, not being appropriate for real-world applications.

### 8.2. Explaining Individual Predictions

In this section, we give a closer look at individual predictions to understand how validation influences at instance level. For this purpose, we present results based on shap force plot [60,64]. The force plot shows shap values contributions in generating the final prediction using an additive force layout. We can visualize feature attributions as “forces”. The prediction starts from the baseline. The baseline for Shapley values is the average of all predictions. In the plot, each Shapley value is an arrow that pushes to increase with positive value (red) or decrease with negative value (blue) the prediction. These forces balance each other out at the actual prediction of the data instance.

We used data from subject 9 from the UCI database to conduct this experiment. For the walking activity, the model obtains high accuracy for cross-validation methodology. These results are expected and also confirm the results presented earlier in this section. For both methodologies, the top five features that contribute negatively to model prediction are the same. In addition, the model tends to give more importance to a set of different features according to the chosen methodology.

**Figure 9:**
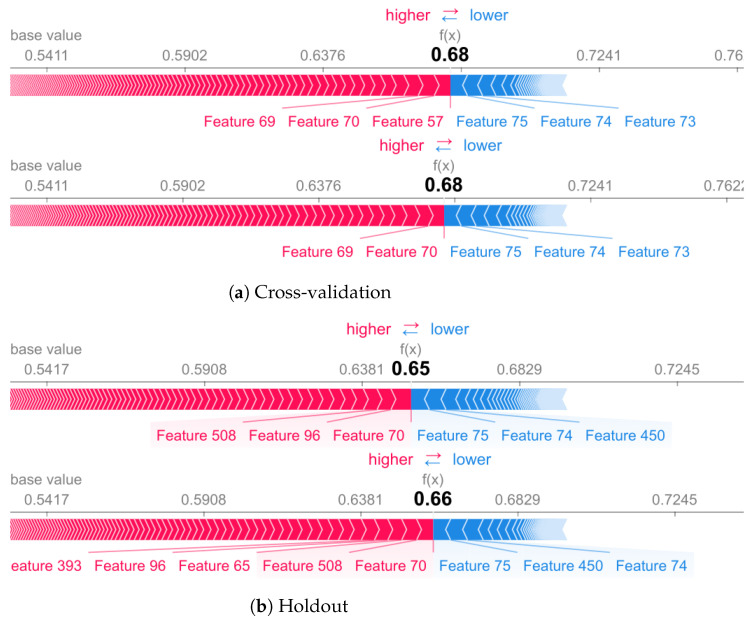
Walk activity for cross-validation (**a**) and holdout (**b**) validation methodology.

As shown in Figure 9b, half of the features (50%), if we look at the top 10 most important, are different for holdout and cross-validation. Feature number 70, based on the accelerometer, is ranked as one of the most important for the walking class. Features such as 393 and 508 are ranked as important when using holdout but do not appear in cross-validation. The cross-validation has features such as number 57, based on the accelerometer energy, which is top-rated by the model.

**Figure 10:**
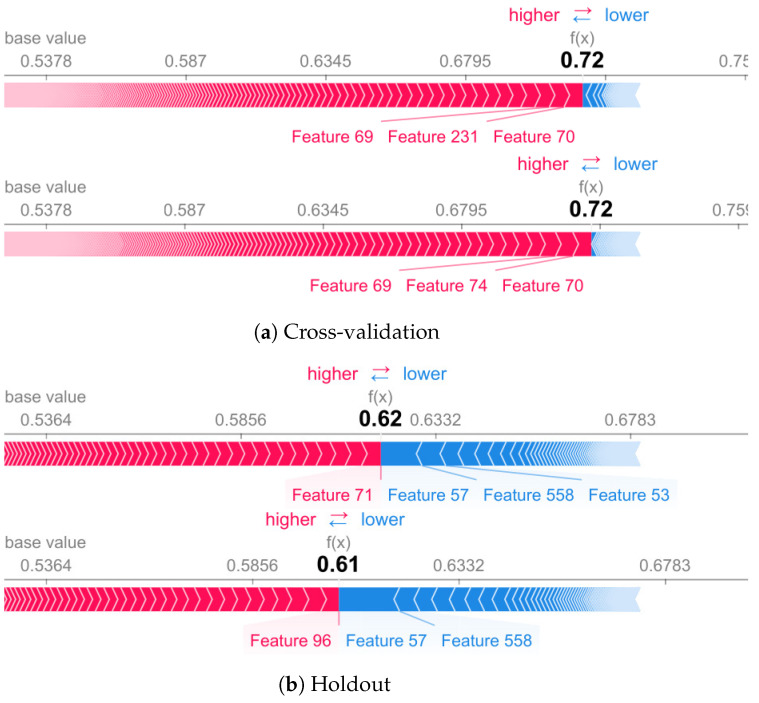
Walking upstairs activity for cross-validation (**a**) and holdout (**b**) validation methodology.

For non-stationary activities, such as walking and walking upstairs (Figure 10), the model shows a greater difference in prediction performance when compared to non-stationary activities. The model accuracy can achieve up to 10% higher when the cross-validation methodology is used. Moreover, the model can pick up to 50% different features to predict user activity when using each methodology.

Figure 10 show that the difference in the model accuracy can achieve up to 10%, showing that the classifier can overestimate the results when he knows patterns of an individual, choosing the features that best represent him. These results for the upstairs activity are similar to the walking activity. For the top 10 features, up to 50% can be different for holdout and cross-validation. Features like 71 are marked as relevant when using holdout for walking upstairs class, but they don’t even appear in cross-validation.

**Figure 11:**
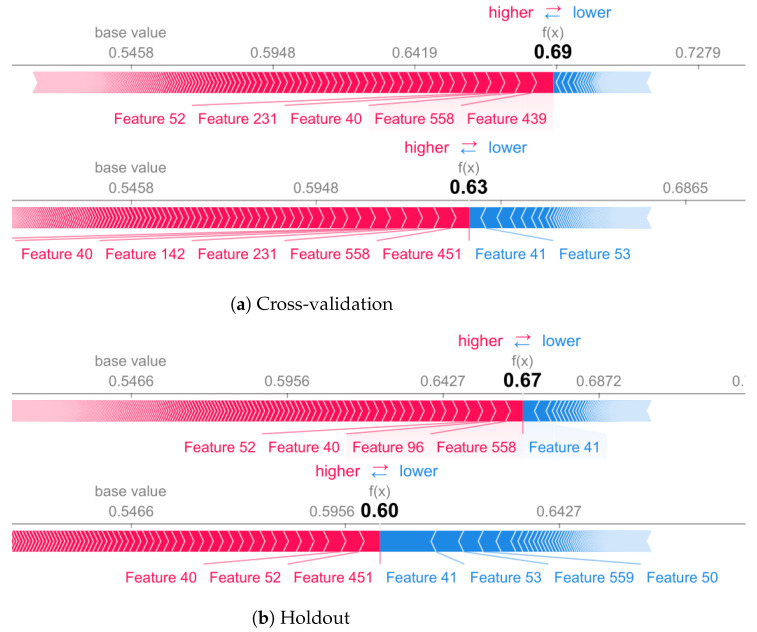
Standing activity for cross-validation (**a**) and holdout (**b**) validation methodology.

**Figure 12:**
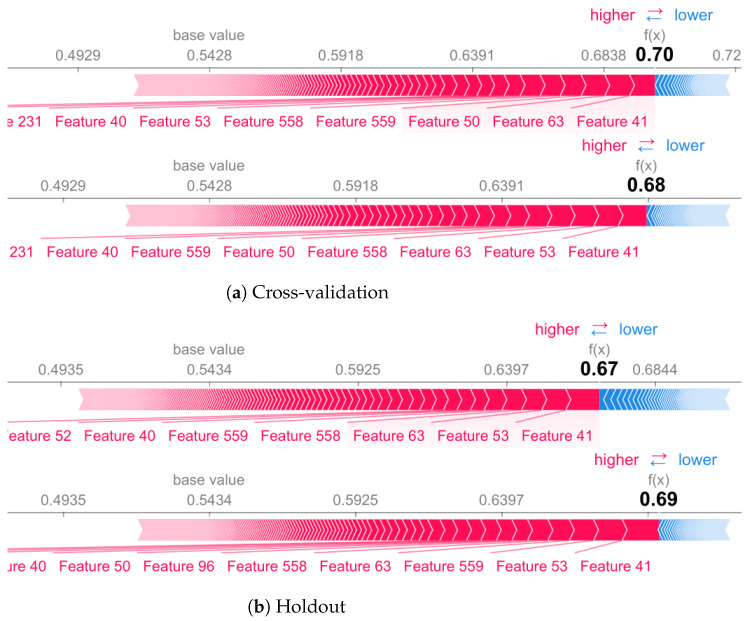
Sitting activity for cross-validation (**a**) and holdout (**b**) validation methodology.

Figure 11 and Figure 12 showed that for stationary activities (e.g., stand activity) the model presents a similar performance in terms of accuracy in both methodologies, CV and holdout. However, there are differences between the selected features for decision making. For standing class, features such as 439 are marked as important when using cross-validation, but they do not appear in holdout (top 10).

We can observe in these studies that, when analyzing the predictions individually for a given class, the classifier can change the order of importance between the features, but we perceive these changes more drastically when holdout and cross-validation are compared.

## 9. Conclusions

In this paper, we present and discuss in detail the importance of a proper evaluation during the design and assessment of a HAR system with inertial sensors. We have conducted several experiments intending to exemplify the overall impact of the evaluation procedures on accuracy and fairness. We also illustrate how these procedures can be set up, showing how to reduce the accuracy overestimation. For this task, the tests were performed in three datasets (UCI-HAR, SHOAIB and WISDM) using *k*-fold cross-validation and leave-one-subject-out validation procedures. The main conclusions drawn from these results are summarized below.

The models that use *k*-CV in the data achieved 98% of accuracy. However, when considering individual information (i.e., the label associated to the subject), the accuracy achieves 85.37% in the best scenario. There is a 12% loss of accuracy when choosing a better evaluation method, that is, the initial result was overestimated by 12%.

The universal model performs poorly in the test phase and also has greater margins of error when compared with personalized models. This shows that the model will struggle to recognize new subjects. In general, the model may perform well in the training phase, but it has a degraded performance in the test set that leads to overfitting. To build a universal model, traditional *k*-CV is not the best solution. For this scenario, the recommended validation procedure would be LOSO or even Holdout when in scenarios were implement LOSO has a significant impact on training.

In personalized models, there is no problem if *k*-CV is used as a validation procedure since, for this type of application, the algorithm should aim at a model that fits the user. In this scenario, the classification algorithms have higher accuracy since the classification model was trained with instances that are very similar to those found in the test set. The very high accuracy values inductee that this is a suitable model for evaluating a customized application for a specific user. Besides, personal models can be trained with dramatically less data.

The hybrid model is used in many related works but they are not very suitable for real-world situations. Most of the commercial HAR applications have preferences for universal or personalized models. The results obtained from this model also present higher accuracy in the classification because some segments that belong to the same subject may be present in both the test set and training set, leading to an overoptimistic result. Again, this does not reflect the true accuracy.

We have shown how the SHAP framework presents itself as a tool that can provide graphical insights into how human activity recognition models manage to achieve their results. Our work has presented manners that can be explored by using explainable algorithms to improve the transparency of creating machine learning models. The SHAP results reinforce that the incorrect choice of validation methodology leads to changes in how attributes are used by models to improve their performance. This situation may cause poor prediction performance and can lead to unreliable results.

Our evaluations also reveal that while current XAI tools provide important functions for data and model analysis, they are still lacking when it comes to analyzing results in scenarios where it is not trivial to find meanings in statistical features extracted from sensors data.

## Figures and Tables

**Table 1:** Summarization of accuracy, recall, precision and F-measure. TP means true positives, TN true negatives, FP false positives and FN means false negatives.

Metric	Equation	Description
Accuracy	$\frac{TP+TN}{TP+TN+FP+FN}$	Accuracy is the ratio of correct predictions divided by the total predictions.
*Precision*	$\frac{TP}{TP+FP}$	*Precision* is the ratio of true positives and total positives predicted.
*Recall*	$\frac{TP}{TP+FN}$	*Recall* is the ratio of true positives to all the positives in ground truth.
*F* Measure	$2\times \frac{Precision\times Recall}{Precision+Recall}$	The *F*-measure is the harmonic mean of *Precision* and *Recall*.

**Table 2:** Summarization of SHOAIB (SH) [2], WISDM [25] and UCI [26] datasets. Items marked with (*) were not used in the experiment.

Dataset	SHOAIB SH	WISDM	UCI
Individuals	10	19 (36)	30
Hz	50	20	50
Segment length	2.5 sec	2.5 sec	2.5 sec
Sensors	Accelerometer, Gyroscope *, Magnetometer *	Accelerometer	Accelerometer, Gyroscope *
Location	Belt, Left Pocket, Right Pocket, Upper Arm, Wrist	Belt	Belt
Activities used	Walking, Running, Sitting, Standing, Walking Upstairs, Walking Downstairs, Jogging *, Biking *	Walking, Jogging, Sitting Standing, Walking Upstairs, Walking Downstairs	Walking, Lying Down, Sitting, Standing, Walking Upstairs, Walking Downstairs

**Table 3:** List of all features used in the experiments with the baseline classifiers.

Domain	Features
Time	min, max, amplitude, amplitude peak, sum, absolute sum, Euclidian norm, mean, absolute mean, mean square, mean absolute deviation, sum square error, variance, standard deviation, Pearson coefficient, zero crossing rate, correlation, cross-correlation, auto-correlation, skewness, kurtosis, area, absolute area, signal magnitude mean, absolute signal magnitude mean, magnitude difference function.
Frequency	Energy, energy normalized, power, centroid, entropy, DC component, peak, coefficient sum.

**Table 4:** Random Forest results for universal model in SHOAIB dataset.

Universal						
User	Accuracy	TP Rate	FP Rate	Precision	Recall	F-Measure
1	84.954	0.850	0.030	0.861	0.850	0.846
2	90.000	0.900	0.020	0.921	0.900	0.900
3	81.759	0.818	0.036	0.837	0.818	0.808
4	84.815	0.848	0.030	0.864	0.848	0.844
5	84.583	0.846	0.031	0.862	0.846	0.847
6	81.019	0.810	0.038	0.812	0.810	0.809
7	85.694	0.857	0.029	0.859	0.857	0.856
8	89.676	0.897	0.021	0.913	0.897	0.894
9	75.417	0.754	0.049	0.800	0.754	0.736
10	83.241	0.832	0.034	0.869	0.832	0.834
Mean	84.116	0.841	0.032	0.860	0.841	0.837
Std. Dev.	4.013	0.040	0.008	0.036	0.040	0.044

**Table 5:** Random Forest results for personalized model in SHOAIB dataset.

Personalized						
User	Accuracy	TP Rate	FP Rate	Precision	Recall	F-Measure
1	97.546	0.975	0.005	0.976	0.975	0.976
2	99.537	0.995	0.001	0.995	0.995	0.995
3	98.056	0.981	0.004	0.981	0.981	0.981
4	99.259	0.993	0.001	0.993	0.993	0.993
5	97.315	0.973	0.005	0.973	0.973	0.973
6	96.759	0.968	0.006	0.968	0.968	0.968
7	97.269	0.973	0.005	0.973	0.973	0.973
8	98.380	0.984	0.003	0.984	0.984	0.984
9	97.500	0.975	0.005	0.975	0.975	0.975
10	98.380	0.984	0.003	0.984	0.984	0.984
Mean	98.000	0.980	0.004	0.980	0.980	0.980
Std. Dev.	0.851	0.008	0.002	0.008	0.008	0.008

## Data Availability

The WISDM can be found on http://www.cis.fordham.edu/wisdm/includes/datasets/latest/WISDM_ar_latest.tar.gz (accessed on 23 May 2019). The SHOAIB dataset can be found in the author’s researchgate profile or https://www.researchgate.net/profile/Muhammad_Shoaib20/publication/266384007_Sensors_Activity_Recognition_DataSet/data/542e9d260cf277d58e8ec40c/Sensors-Activity-Recognition-DataSet-Shoaib.rar (accessed on 12 February 2020). The UCI-HAR dataset is avaliable on https://archive.ics.uci.edu/ml/machine-learning-databases/00240/UCI%20HAR%20Dataset.zip (accessed on 22 May 2019).

## Abbreviations

The following abbreviations are used in this manuscript:

ADL	Activity Daily Living
ARP	Activity Recognition Process
CNN	Convolutional Neural Networks
DL	Deep Learning
HAR	Human Activity Recognition
i.i.d.	Independent and Identically Distributed
*k*-CV	*k*-fold Cross-Validation
LOOCV	Leave-One-Out cross-Validation
LOOSO	Leave-One-Subject-Out
LSTM	Long-Short-Term Memory Recurrent
ML	Machine Learning
SHAP	Shapley additive explanations
XAI	Explainable Artificial Intelligence
